# ocsESTdb: a database of oil crop seed EST sequences for comparative analysis and investigation of a global metabolic network and oil accumulation metabolism

**DOI:** 10.1186/s12870-014-0399-8

**Published:** 2015-01-21

**Authors:** Tao Ke, Jingyin Yu, Caihua Dong, Han Mao, Wei Hua, Shengyi Liu

**Affiliations:** Key Laboratory for Oil Crops Biology, the Ministry of Agriculture, PR China, Oil Crops Research Institute, Chinese Academy of Agricultural Sciences, No.2 Xudong Second Road, Wuhan, 430062 China; Department of Life Science and Technology, Nanyang Normal University, Wolong Road, Nanyang, 473061 China

**Keywords:** Database, Expressed sequence tag, Metabolic network, Oil crop seeds

## Abstract

**Background:**

Oil crop seeds are important sources of fatty acids (FAs) for human and animal nutrition. Despite their importance, there is a lack of an essential bioinformatics resource on gene transcription of oil crops from a comparative perspective. In this study, we developed ocsESTdb, the first database of expressed sequence tag (EST) information on seeds of four large-scale oil crops with an emphasis on global metabolic networks and oil accumulation metabolism that target the involved unigenes.

**Description:**

A total of 248,522 ESTs and 106,835 unigenes were collected from the cDNA libraries of rapeseed (*Brassica napus*), soybean (*Glycine max*), sesame (*Sesamum indicum*) and peanut (*Arachis hypogaea*). These unigenes were annotated by a sequence similarity search against databases including TAIR, NR protein database, Gene Ontology, COG, Swiss-Prot, TrEMBL and Kyoto Encyclopedia of Genes and Genomes (KEGG). Five genome-scale metabolic networks that contain different numbers of metabolites and gene–enzyme reaction–association entries were analysed and constructed using Cytoscape and yEd programs. Details of unigene entries, deduced amino acid sequences and putative annotation are available from our database to browse, search and download. Intuitive and graphical representations of EST/unigene sequences, functional annotations, metabolic pathways and metabolic networks are also available. ocsESTdb will be updated regularly and can be freely accessed at http://ocri-genomics.org/ocsESTdb/.

**Conclusion:**

ocsESTdb may serve as a valuable and unique resource for comparative analysis of acyl lipid synthesis and metabolism in oilseed plants. It also may provide vital insights into improving oil content in seeds of oil crop species by transcriptional reconstruction of the metabolic network.

**Electronic supplementary material:**

The online version of this article (doi:10.1186/s12870-014-0399-8) contains supplementary material, which is available to authorized users.

## Background

Oil crop seeds are important sources of fatty acids (FAs) and proteins for human and animal nutrition as well as for non-dietary uses [1]. As a major goal of oil crop seed research, studies focusing on engineering seeds with enhanced oil quantity and quality has prompted efforts to better understand the processes involved in seed metabolism, especially in the accumulation of storage products [2]. There are four major oil crops with different oil content in seeds: rapeseed (*Brassica napus*), soybean (*Glycine max*), sesame (*Sesamum indicum*) and peanut (*Arachis hypogaea*). Accumulation levels of seed storage compounds, such as triacylglycerol (TAG), proteins and carbohydrates, show significant species-specific variations. Sesame and peanut have heterotrophic oilseeds (non-green oilseeds) that contain up to 60% FAs of dry seed, whereas soybean and rapeseed have autotrophic oilseeds (green seeds) that contain up to 20% and 40% FAs of dry seed, respectively [3]. Although non-green seeds of sesame and peanut can accumulate oil without the benefit of photophosphorylation, they have the highest oil content among oilseeds. This suggests that there are many differences in terms of carbon flow, carbon recapture and ATP and NADPH production between non-green seeds and green seeds [4,5].

cDNA and/or genome sequence data of these important oil crops are becoming publicly available. To date, the soybean reference genome has been released. Progress has been made in genome sequencing projects for peanut (the international peanut genome initiative [IPGI]) and sesame [6,7] and Brassica napus [8]. Large-scale expressed sequence tag (EST) collections are also making valuable contributions to the investigation of genetic traits of crops. More than 2,328,985 EST sequence entries are available in the public database (dbEST database of NCBI, as of October 2013) for the important oil crops *B. napus* (643,944), *G. max* (1,461,723), *S. indicum* (44,820) and *A. hypogaea* (178,498). However, these huge data sets are under-utilised due to the scarcity of informatics databases. A handful of such informatics resources are currently available, which provide high-level analysis of crop functional genomics in searchable forms [9-11].

Genome-scale metabolic network models have been successfully used to describe metabolic processes in various microbial organisms [12]. These system-based frameworks enable systematic biological studies and have the potential to contribute to metabolic engineering. Reconstruction of a complete, genome-scale metabolic network is usually based on annotated genomic sequences [13], but the activities of many proteins and enzymes are highly tissue-specific, and therefore, metabolic networks should be tissue-specific as well [14]. The biochemical pathways and metabolisms in specific plant tissues are more complicated than those in bacteria. For example, during the development of oilseeds, the synthesis of large quantities of stored TAG relies on sucrose and hexose transport from the mother plant. Recent studies have revealed that a broad range of metabolites are taken up and utilised by plastids for FA synthesis; this process depends on the plant species, organs and stage of development [15]. As a result, whole genome metabolism network construction of oilseeds from multiple oil crops in specific developmental stages is quite essential as well as based on the whole genome data. Based on such a resource, protein coding sequences (CDSs) can be identified, annotated by Enzyme Commission (EC) numbers, and linked to specific biochemical reactions. The reactions can then be connected and further interpreted as a network and analysed using the Cytoscape program [16].

Some comprehensive repositories of plant resources have been established. PlantGDB is a popular site for plant genomic and EST data. This site provides tools and data of plant EST assemblies and genome annotation [17]. Plant Metabolic Network (PMN) consists of plant metabolic pathway databases [18] (http://www.plantcyc.org/). In recent years, numerous studies on oilseed development and lipid metabolism have integrated extensive data sets. Most studies have focused on the model plant *Arabidopsis thaliana* and have included projects such as Microarray Analysis of Developing Arabidopsis Seeds [19-21], ARALIP: Arabidopsis Acyl-Lipid Metabolism [22], and Quizzing the Chemical Factories of Oilseeds (NSF-Plant Genome Grant) (http://bioinfolab.unl.edu/oilseeds/databases.html). Each of these databases and websites not only provide information on Arabidopsis seed lipid metabolism and the network of gene expression during Arabidopsis seed filling but also include EST data and seed transcriptional profiling data of some other oilseed species.

The ‘-omics’ data of oil crops in publicly available databases are usually far from comprehensive and integrated. Comparative analyses between oil crop tissues to identify species- or tissue-specific genes involved in lipid and oil metabolic are absent. Also, there is a lack of databases that assemble oil crop species together with annotations based on comparative genomics. To understand molecular metabolism involved in oil crop propagation, the accumulation of a storage product and oil biosynthesis, we collected EST sequences on a large scale from seeds at different developmental stages for rapeseed, soybean, sesame and peanut. To understand the EST sequence and full-length CDSs of seeds of four oil crops and to facilitate research on comparative metabolic networks, we constructed a new database called ‘ocsESTdb’ (oil crop seed EST database) with seed EST sequences and metabolic networks of four oil crop species with different objectives. The first objective is to provide large-scale EST sequences and complete amino acid sequences from full-length CDSs and to provide information on clusters, annotations and pathways. The second objective is to provide comparative annotations of four oil crops and metabolic pathways. The third objective is to develop a genome-scale metabolic network model based on the large-scale sequencing of oilseeds at different developmental stages. The ocsESTdb database integrates knowledge of seed EST sequences and full-length CDSs of four oil crops seeds and reconstructs of the metabolic network with insights into comparative oil crop genomics. ocsESTdb can be accessed via the Web interface at http://www.ocri-genomics.org/ocsESTdb/.

## Construction and content

### Implementation

**Figure 1:**
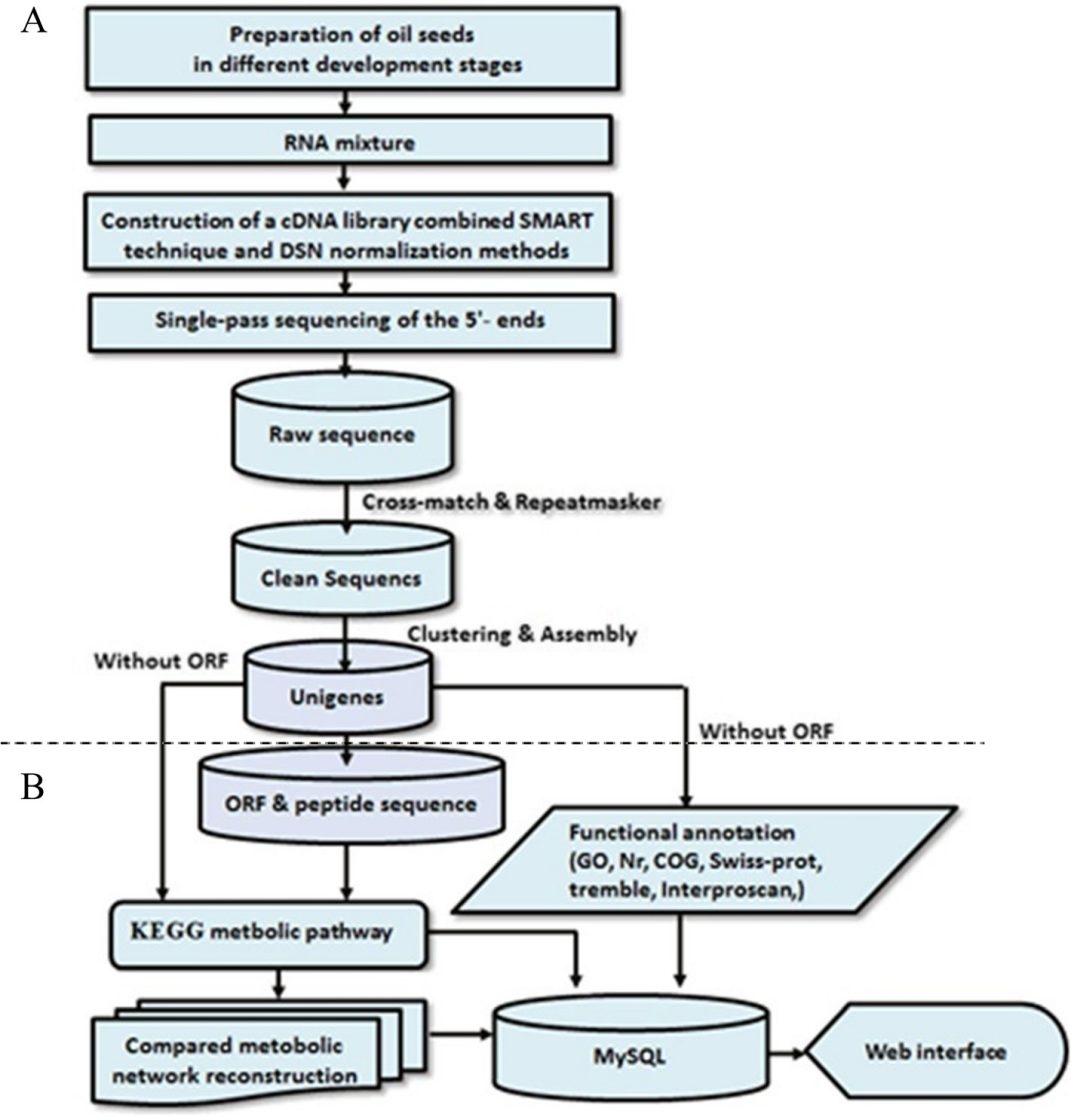
**Schematic representation of the informatics workflow used to generate ocsESTdb and related annotation. (A)** Part of this workflow is described as a procedure for processing ESTs raw data of oilseeds at different developmental stages. **(B)** Part of this workflow is the pipeline of unigene annotation in ocsESTdb as well as the procedure of developing ocsESTdb.

The ocsESTdb database was developed using Perl/CGI, Python and JavaScript on a platform with the Apache Web server on CentOS 5.4 and the MySQL 5.0 database management system. We developed a pipeline (Figure 1) to organise data. The pipeline provides a schematic of the steps involved in data processing and database construction. For the schematic of steps in database construction, the pipeline is composed of a series of fully integrated, open resource software and will automatically process, analyse and import data into a MySQL database management system. Major modules of the database construction pipeline include the following three steps. First is to process data for ocsESTdb: logical relationships among sequence data, annotation information, pathways and networks are constructed based on unigenes assembled with clean raw ESTs. Second is to prepare the MySQL database for ocsESTdb: unigenes serve as primary keys in the data table to create logical relationships among the data tables in MySQL database. Third is to visualise the interface for ocsESTdb: a Web-based, searchable and downloadable database is constructed to provide a high-level data resource on processed sequence information, functional annotation and biological meaning assigned to total clean raw EST sequences based on logical relationships. The ocsESTdb database is divided into different sections to satisfy different functional needs and to provide users with the flexibility to access, search and download all analysis results separately.

### Data source

Combined with SMART techniques (Clontech), three normalized cDNA libraries enriched in full-length sequences were constructed for the generation of ESTs by using mRNA isolated from immature seeds of three high-oil content cultivars (soybean, peanut and sesame) at three prominent different oil accumulation stages after pollination [23-25]. The cDNA library of *B. napus* was constructed from immature seeds of two rapeseed lines, *B. napus* cv. ZY036 (high-oil contents, HO) and *B. napus* cv. 51070 (low-oil contents, LO), by 454 sequencing (2 weeks after flowering) [26] (Table 1).

**Table 1:** **Raw data source for EST sequences**

**Species**	**Representative**	**Database**	**Series of Seq.**
*Arachis hypogaea*	Ahy	EST-NCBI	JK146921-JK167516
*Brassica napus*	Bna	SRA-NCBI	SRX006869, SRX006870
*Glycine max*	Gma	EST-NCBI	HO008567-HO045222
*Sesamum indicum*	Sin	EST-NCBI	JK045130–JK086377

### Raw data processing and clustering analysis

Combined with SMART techniques (Clontech), three normalised cDNA libraries enriched in full-length sequences were constructed for the generation of ESTs using mRNA isolated from immature seeds of three high-oil content cultivars (soybean, peanut and sesame) at three prominent different oil accumulation stages after pollination [23-25]. The cDNA library of *B. napus* was constructed from immature seeds of two rapeseed lines, *B. napus* cv. ZY036 (high-oil content, HO) and *B. napus* cv. 51070 (low-oil content, LO) by 454 sequencing (2 weeks after flowering) [26] (Table 1). Quality control of raw DNA sequences was performed by using Phred program [27] to remove sub-standard reads, the vector and adapter sequences, followed by EST-trimmer (http://pgrc.ipk-gatersleben.De/misa/download/est_trimmer.pl) to eliminate 3' polyA and 100 bp EST reads. After screening of low-quality DNA and trimming of vector sequences, Phrap program was used to cluster the overlapping ESTs into contigs [27]. Groups that contained only one sequence were classified as singletons.

### Comprehensive annotation of oil crop unigenes

ESTs and unigenes were translated into six reading frames and analysed against the Arabidopsis genomic databases TAIR (http://www.arabidopsis.org/), UniProtKB/Swiss-Prot, UniProtKB/TrEMBL and GenBank NR using the default setting of BLASTX program (NCBI, ftp://ftp.ncbi.nlm.nih.gov/blast). The results of BLASTX and BLASTN with E-values equal to or less than 10^−5^ were treated as ‘significant matches’, whereas ESTs with no hits or matches and with E-values more than 10^−5^ were treated as ‘no significant matches’. ESTs and unigenes were annotated according to the top BLASTX match (Table 2). The results were then parsed and stored in the ocsESTdb database. Using the same protocols, the unigene/EST sequences were further annotated using the COG database (http://www.ncbi.nlm.nih.gov/COG/) [28] and the Kyoto Encyclopedia of Genes and Genomes (KEGG) pathway database [29].

**Table 2:** **Summary of expressed sequence tags (ESTs) from the five oil crops seed cDNA libraries**

**Categories**	**No. of sequences generated**	**No. of high-quality sequences**	**Average size of high-quality sequences (bp)**	**Singletons**	**Contigs**	**No. of unigene**	**No. of ORF**	**Unabundancy**	**full length gene (%)**
*A. hypogaea*	21,998	20,596	579	13,678	2,040	15,718	14,906	66.4%	57.0%
*B. napus* (HO)	78,332	69,938	216	14,582	7,130	21,712	7,658	20.9%	
*B. napus* (LO)	57,870	50,656	213	9,734	4,165	13,899	4,028	19.2%	
*G. max*	44,753	36,656	624	24,245	3,737	27,982	26,878	66.1%	55.0%
*S. indicum*	45,569	41,248	570	23,863	3,661	27,524	25,043	57.9%	45.0%
Total	248,522	219,094		86,102	20,733	106,835	78,513		

GO annotations of all the seed unigenes were performed using the Blast2GO program [30] (Table 3). These sequence libraries had different significant matches ratios to sequences in TAIR, the non-redundant protein database (Nr), Swiss-Prot and TrEMBL database based on an E value cut off which was equal or less than 10–5. A total of 62,220 unigenes were successfully annotated with GO terms. The lowest annotation ratio is rape most attributed to the more short sequence from the 454 sequence method. This software performed a BLASTX similarity search against the GenBank non-redundant protein database, retrieved GO terms for the top 12 BLAST results, and annotated the sequences based on defined criteria. Additional information on unigenes was obtained using InterProScan [31,32] and KEGG (KO number and EC number) annotation, and additional sequences were then annotated. GO enrichment analysis was compared and visualised using WEGO (http://wego.genomics.org.cn) [33].

**Table 3:** **Statistics of annotation result for unigenes from the five oil crops seed cDNA libraries**

**Species**	**No. of unigenes**	**Hit to A. thaliana (%)**	**Hit to Nr (%)**	**Hit to Swiss-Prot (%)**	**Hit to TrEMBL (%)**	**Annotated to GO (%)**
*B. napus* (LO)	13899	7681(55.3%)	5377(38.7%)	3638(26.2%)	5336(38.4%)	1870(13.5%)
*B. napus* (HO)	21712	13076(60.2%)	9925(45.7%)	6733(31.0%)	9293(42.8%)	3358(15.5%)
*A. hypogaea*	15718	12618(80.3%)	13561(86.3%)	10714(68.2%)	13479(85.8%)	13114(83.4%)
*G. max*	27982	24439(87.3%)	25090(89.7%)	20745(74.1%)	26880(96.1%)	25403(90.8%)
*S. indicum*	27524	15236(55.4%)	21193(77.0%)	15797(57.4%)	27173(98.7%)	18475(67.1%)
Total	106835	73050	75146	57627	82161	62220

### Reconstruction of a global metabolic network of four oil crops

**Figure 2:**
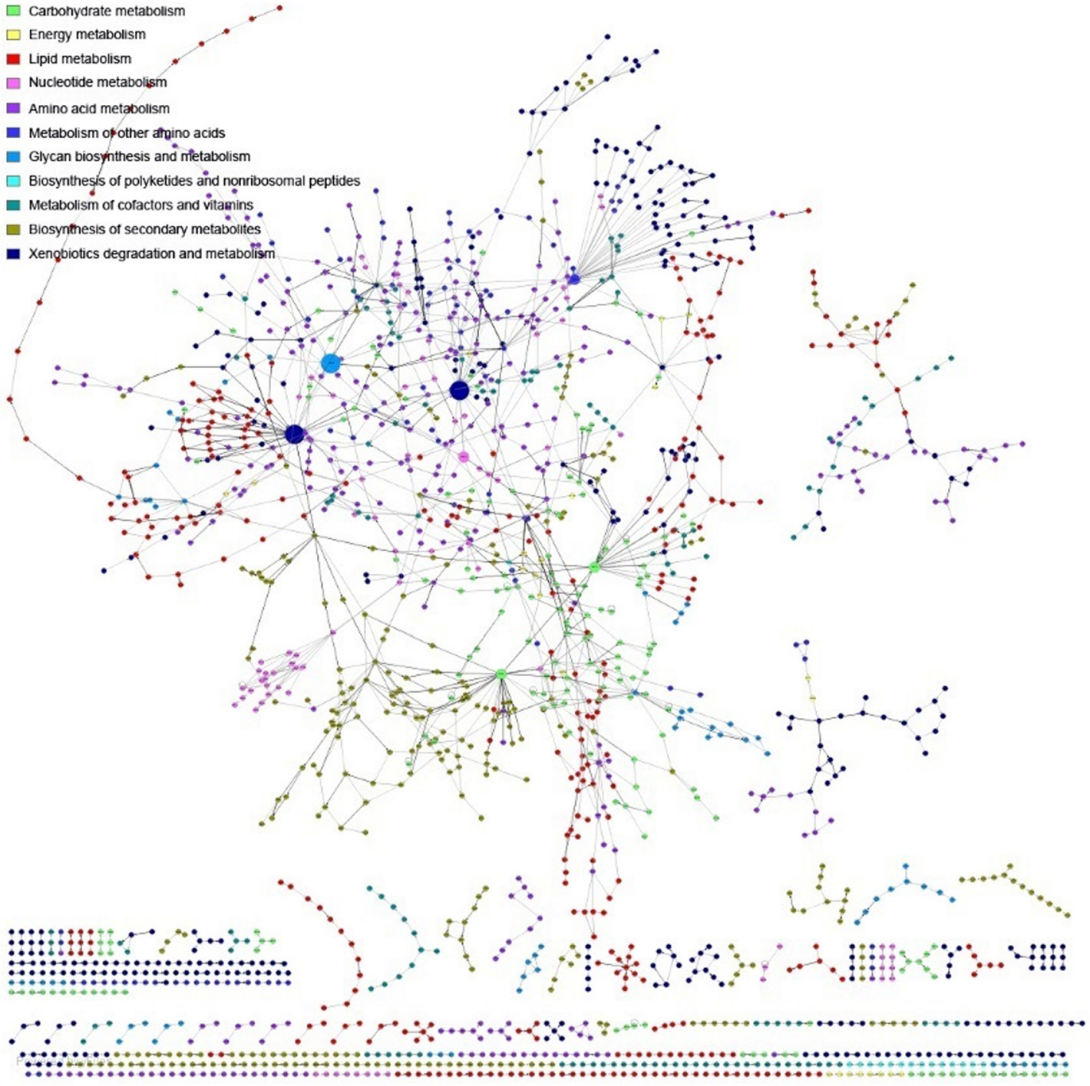
**The genome-scale metabolic network of** ***Arachis hypogaea.*** Nodes are metabolites whereas links are reactions. The colours of the nodes represent different functional categories. The sizes of the nodes are proportional to the number of reactions from or to that node (metabolite) in the genome-wide network. For a detailed and clickable version, see the net pages in the database.

A metabolic network was constructed based on the list of enzymes, especially EC numbers, which were extracted from the unigene annotation and the corresponding reactions of which were acquired by searching in an established biochemical reaction database [13,34]. Biochemical reactions were then connected to each other with metabolics as the node and reactions as the edges. The reaction database was based on the KEGG LIGAND database (http://www.genome.jp/ligand). The organism-specific metabolic networks were reconstructed from the enzyme–gene relation and the reaction–enzyme information. The connection matrix of reactions [34,35] was substantially improved in this work by updating the enzyme reaction database to the newer version of KEGG Ligand (Status August 2009). Finally, the new version contained 7,908 reactions compared with the 6,442 reactions in the former version (Additional file 1: ReactionDB_20090524.xls). The programs Cytoscape [36] (http://www.cytoscape.org) and yEd (a Java Graph Editor from the company yWorks) (http://www.yworks.com) were used as layout tools for the genome-wide network. We constructed metabolic networks of five oil crop seeds. The metabolic network of peanut (Figure 2) has the highest number of biological reactions and metabolites, containing 2337 biological reactions and 1923 metabolites.

## Utility

The ocsESTdb database provides a user-friendly interface that is divided into five main functional tabs: ‘Home’ , ‘Browse’ , ‘Search’ , ‘Document’ and ‘Help’. Each functional tab provides a specific capability for users to retrieve information on oil crop seed ESTs or unigenes from the database or to view the oil crop seed ESTs or unigenes in the context of participating in the either the acyl-lipid metabolism pathways or networks constructed by metabolites of oil crop seed unigenes.

### Major friendly interface provided by ocsESTdb

**Figure 3:**
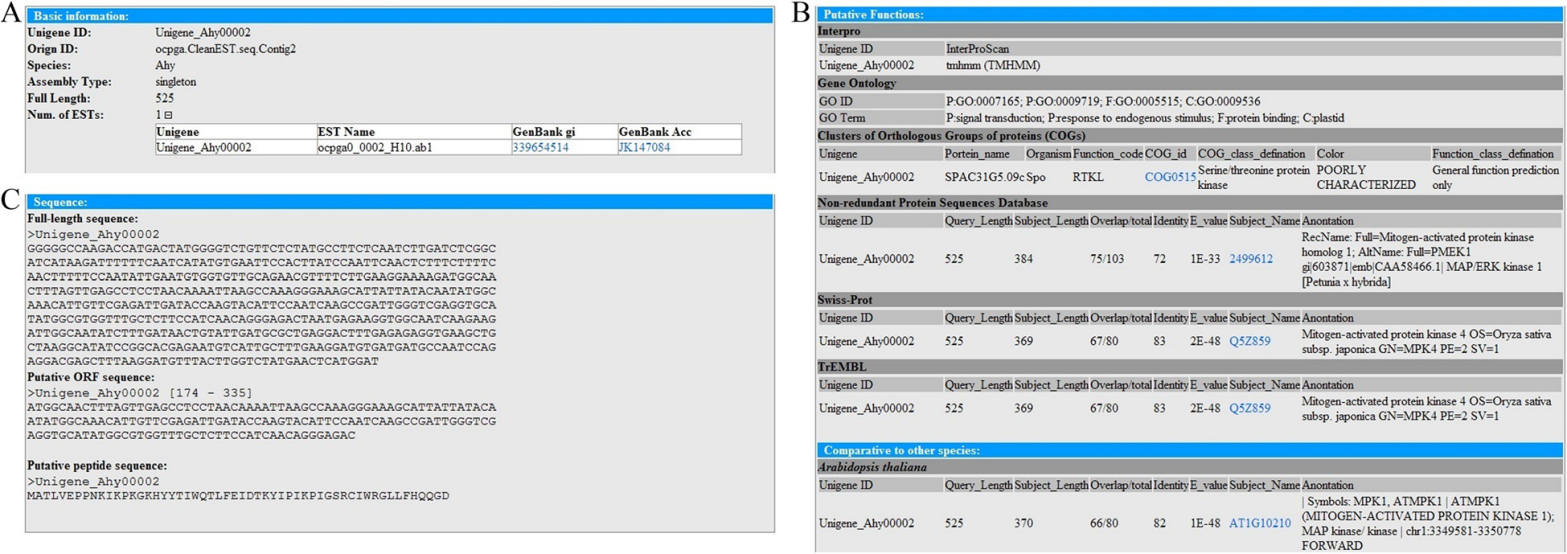
**Typical example of the detailed annotation of ocsESTdb unigenes. (A)** Basic information on unigenes in ocsESTdb, including the unigene type and ESTs information that clusters the corresponding unigenes; **(B)** Annotation information on unigenes in ocsESTdb, including conserved domains predicted by InterProScan, list of GO terms, best hit in Clusters of Orthologous Groups of proteins, Non-redundant Protein Sequences Database, Swiss-Prot, TrEMBL as well as comparisons to the model species *Arabidopsis thaliana*. Especially, for unigenes of *Glycine max*, comparative analysis with gene data sets of the *G. max* genome was added in this section; **(C)** Nucleotide sequence and deduced open reading frame (ORF) sequence and peptide sequence for unigene in ocsESTdb.

**Figure 4:**
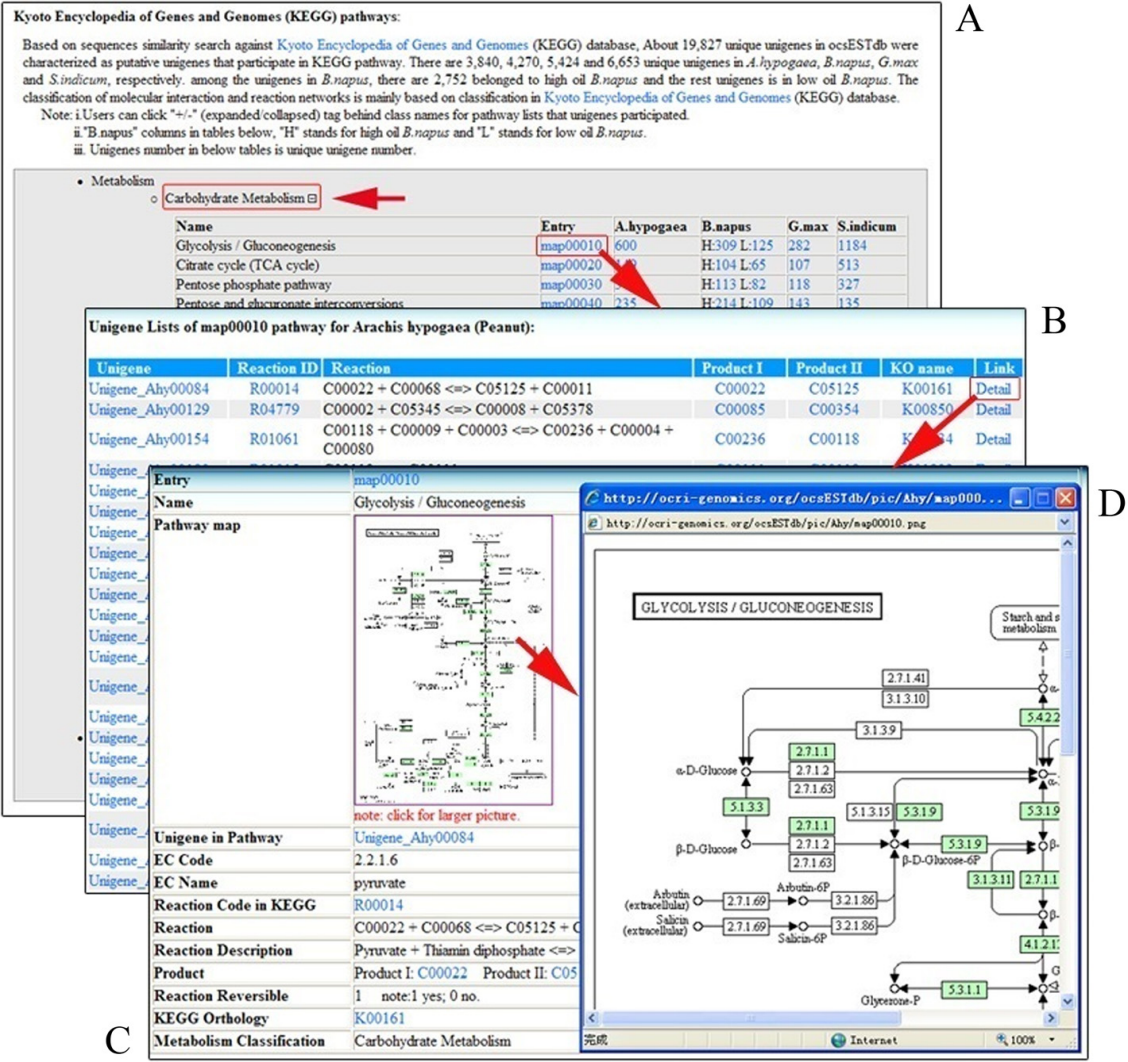
**Typical example of a KEGG pathway that four oil crop seed unigenes participated in. (A)** Lists of putative pathways that ocsESTdb unigenes referred to. **(B)** Lists of putative reactions that ocsESTdb participated in, including reaction, metabolites and KEGG orthology. **(C)** Detailed pathway information that ocsESTdb unigenes referred to, including KEGG entry, reaction code in KEGG database, reaction, metabolites, KEGG orthology and metabolism classification. **(D)** KEGG pathway.

The ‘Browse’ tab contains three functional units: species, pathway and network. The ocsESTdb database supplies convenient browse functions to help users retrieve statistics of raw EST sequences, clustering reads, assembling contigs and unigenes, unigene list, and gene ontology enrichment analysis for different oil crop species. Users can see all assembled unigenes by clicking the hyperlink of unigene lists and can then obtain the comprehensive annotation of unigenes for this species by clicking the unigene’s name. For each unigene, this database also offered a useful interface to allow users to download cleaned ESTs for corresponding unigene by referring to DFCI Gene Index (http://compbio.dfci.harvard.edu/tgi/). The ocsESTdb database supplies a comprehensive annotation on the unigene detail page, including unigene basic information, functional annotation and sequence information (Figure 3). Through the Browse functional section, users can obtain the pathway information on unigenes that participate in different pathways of the four oil crop species. The pathways in ocsESTdb were classified into two types to detect the functions of lipid metabolic pathways for oil crop seed unigenes. One type was curated by Arabidopsis acyl-lipid metabolic pathways, and the other type was based on KEGG pathways. According to different contents of pathways, ocsESTdb collects all unigenes that participate in different pathways and allows the users to make further comparative analyses. Through user-friendly and efficient browsing capabilities of the database, users can obtain information on unigenes involved in different species (Figure 4).

In the network, different colours represent different metabolic types, and each node point represents the meridians involved in the metabolism of possible metabolites.

The ocsESTdb database also supplies a pipeline of data processing and database construction, statistics of data collected in this database, literature and open resource in this field. Users can employ the ‘Help’ functional unit to access and download data of EST and unigene sequences, annotations and pathways.

### General search in database by names or identifiers

The ocsESTdb database provides a full-featured searching function. The user can retrieve information of interest from the search module. Users can obtain detailed annotation information on the target unigene by entering the ID of the specific unigene and corresponding type of unigenes from different species by entering the relevant GO terms, InterPro entry or COG ID. For further comparative research and analysis, users can determine unigenes participating in different pathways of different species by entering the target pathway entry.

### Searching sequence similarity using BLAST

To implement the sequence similarity searching function, ocsESTdb supplies a customised BLAST search from standard NCBI BLAST module for users to retrieve similar or identical sequences from the database with different interests. Users can offer nucleic acid or amino acid sequence via directly pasting or file uploading to match against the oil crop seed ESTs or unigenes database from *B. napus*, *G. max*, *S. indicum* and *A. hypogaea*. Through comparisons using the BLAST search, users can get the annotations of their query sequences with the deposited data in ocsESTdb quickly.

## Discussion

ocsESTdb collects oil crop seed ESTs and unigenes from *B. napus*, *G. max*, *S. indicum* and *A. hypogaea* and supplies a public resource for researchers to comparatively analyse and investigate oil accumulation metabolism. Analyses of these four oilseed EST sets have helped to identify similar and different gene expression profiles during seed development. The BLAST and annotation results could be chosen as an example to comparatively analyse the differences in functional genes between four oilseeds. The comparative results of COG annotation and functional genes of four oilseeds can be found at the ‘Statistics’ pages. There is an obvious difference in ratio of functional category between the green and non-green seeds, especially in the metabolism-related gene category. Two non-green seeds (sesame and peanut) have the same ratios in all categories. The ‘metabolism’ category of the non-green seeds of the two crop species was approximately two times higher than that of soybean seeds. Four oil crops have similar ratios of ‘lipid transport and metabolism’.

A comparison of the seed metabolic networks was also performed. Most of the reactions are connected to the central metabolism in almost the same ratios among the four oilseeds, such as carbohydrate metabolism, amino acid metabolism, lipid metabolism and energy metabolism (Table 4). Acetyl-CoA and pyruvate belong to the metabolites with the highest connectivity in the five metabolic networks. The sesame metabolic network comprises 36 and 41 biochemical reactions involved in Acetyl-CoA and pyruvate, respectively (Table 5).

**Table 4:** **Distribution of reactions of the inferred genome-wide metabolic network in different functional categories**

	***G. max***		***B. napus*** **(LO)**		***B. napus*** **(HO)**		***A. hypogaea***		***S. indicum***	
**Functional category**	Reactions	Metabolites	Reactions	Metabolites	Reactions	Metabolites	Reactions	Metabolites	Reactions	Metabolites
**Carbohydrate Metabolism**	319(13.7%)	269(13.8%)	183(14.4%)	168(10.7%)	221(14.3%)	261(14.5%)	323(13.8%)	275(14.3%)	316(15.65%)	303(13.97%)
**Energy metabolism**	52(2.2%)	69(3.5%)	28(2.2%)	72(4.6%)	33(2.1%)	79(4.4%)	51(2.2%)	66(3.4%)	29(1.44%)	52(2.40%)
**Lipid metabolism**	467(20.1%)	412(21.1%)	246(19.3%)	340(21.7%)	280(18.1%)	361(20%)	478(20.5%)	408(21.2%)	350(17.34%)	391(18.03%)
**Nucleotide metabolism**	170(7.3%)	125(6.4%)	62(4.9%)	88(5.6%)	122(7.9%)	115(6.4%)	124(5.3%)	107(5.6%)	150(7.43%)	120(5.53%)
**Amino Acid metabolism**	380(16.3%)	367(18.8%)	230(18.1%)	336(21.5%)	291(18.8%)	384(21.3%)	437(18.7%)	344(17.9%)	373(18.47%)	432(19.92%)
**Metabolism of other amino acids**	91(3.9%)	120(6.1%)	42(3.3%)	106(6.8%)	54(3.5%)	126(7%)	92(3.9%)	45(2.3%)	72(3.57%)	122(5.62%)
**Glycan biosynthesis and metabolism**	71(3%)	95(4.9%)	37(2.9%)	89(5.7%)	43(2.8%)	94(5.2%)	57(2.4%)	122(6.3%)	85(4.21%)	143(6.59%)
**Biosynthesis of polyketides and nonribosomal peptides**	8(0.3%)	17(0.9%)	8(0.6%)	19(1.2%)	8(0.5%)	19(1.1%)	8(0.3%)	86(4.5%)	10(0.50%)	20(0.92%)
**Metabolism of cofactors and vitamins**	131(5.6%)	180(9.2%)	64(5%)	133(8.5%)	95(6.1%)	176(9.8%)	121(5.2%)	17(0.9%)	141(6.98%)	194(8.94%)
**Biosynthesis of secondary metabolites**	324(13.9%)	332(17%)	222(17.4%)	303(19.3%)	235(15.2%)	327(18.1%)	327(14%)	508(26.4%)	203(10.05%)	287(13.23%)
**Xenobiotics biodegradation and metabolism**	320(13.7%)	399(20.4%)	151(11.9%)	284(18.1%)	164(10.6%)	304(16.9%)	319(13.6%)	384(20%)	289(14.31%)	421(19.41%)
**Total**	**2333**	**1954**	**1273**	**1565**	**1546**	**1803**	**2337**	**1923**	**2019**	**2169**

**Table 5:** **Pathway and unigenes statistics of five oilseeds metabolic network**

	**Unigenes in total pathway**	**Unigene in fatty acid metabolism pathway**	**Reactions involved in Acetyl-CoA**	**Reactions involved in pyruvate**
*A. hypogaea*	3481	639	26	24
*B. napus* (HO)	2753	333	35	27
*B. napus* (LO)	1519	196	22	22
*G. max*	5425	710	25	24
*S. indicum*	5234	1285	36	41

**Figure 5:**
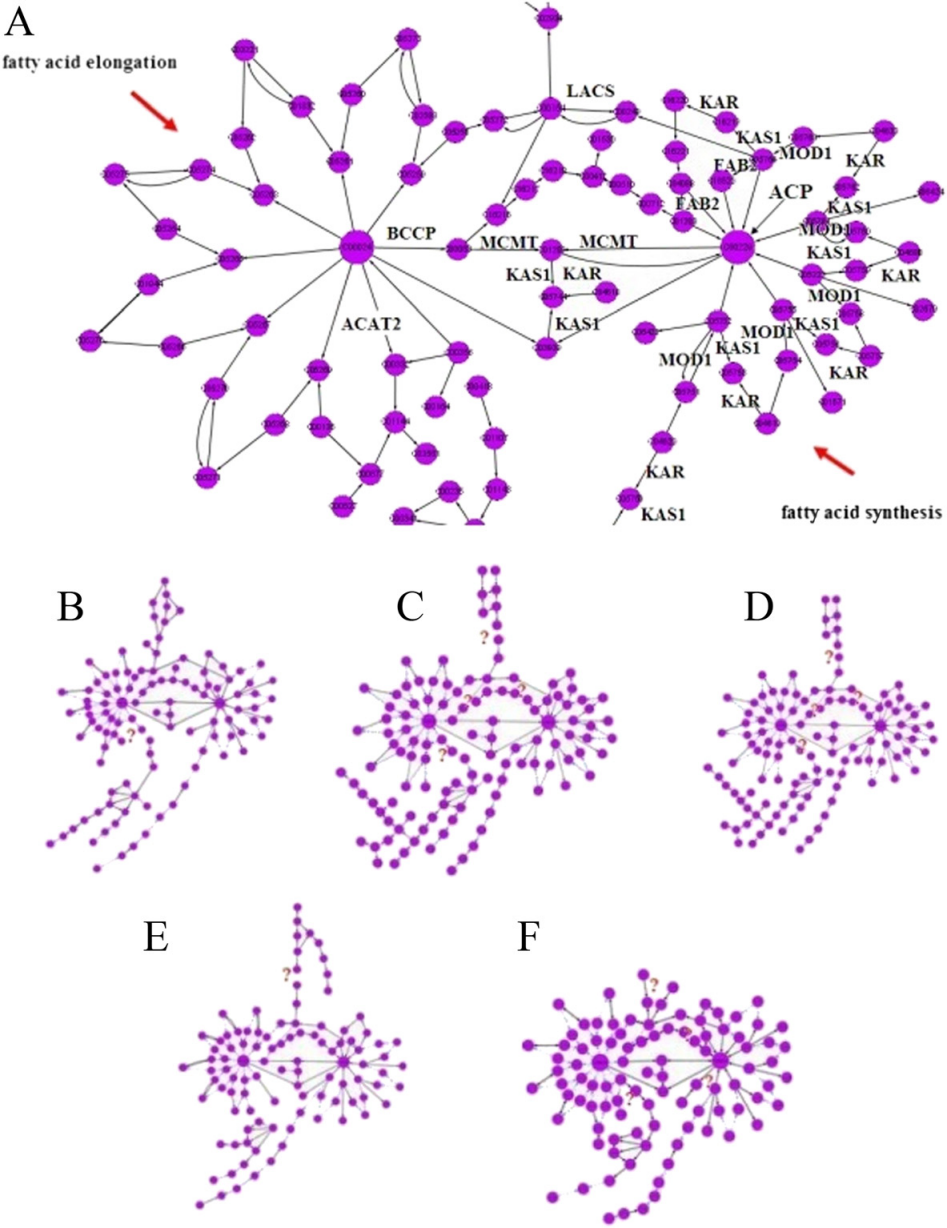
**Network of fatty acid synthesis and fatty acid elongation. (A)** Important enzymes involved in FA synthesis sub-networks; **(B)** peanut (*A. hypogaea*); **(C)** rapeseed (*Brassica napus*) HO; **(D)** rapeseed (*B. napus*) LO; **(E)** soybean (*G. max*) and **(F)** sesame (*Sesamum indicum*).

FAs and oils are the main accumulation products of these four oil crops, and their biosynthesis is crucial to the development of oil crop seeds. Thus, the pathways related to FA biosynthesis and elongations were extracted from the genome-wide metabolic network directly connected to these pathways to compare the different network structures of the four seeds. Among the total 106,835 unigenes in the cDNA data of oil crop seeds, 333, 196, 710, 1,285 and 639 are related to lipid metabolism in high-oil content rapeseed, low-oil content rapeseed, soybean, sesame and peanut, respectively (Table 5). Structural disparity among the seeds of four crops was analysed based on annotated pathways. The FA synthesis sub-networks of five seeds have similar structures (Figure 5). However, small differences can still be noted between them (reactions marked as red in Figure 5).

## Conclusions

The ocsESTdb database is the first integrated comparative analysis database of EST sequences from the seed of four oil crops. This database supplies a user-friendly interface, in which data can be freely accessed and downloaded. ocsESTdb is a uniquely comprehensive world-wide oil crop seed EST database, which also includes sufficient information on unigenes that represent the characteristics of oil crops in terms of oil content. Information necessary to investigate the properties of oil crop genes at the molecular and function levels is also supplied in the database. Moreover, the ocsESTdb database is a tool for information retrieval, visualisation and management. The large set of full-length cDNA clones from oil crops reported in this study will serve as a useful resource for gene discovery and will aid in the precise annotation of the oil crop genome. In addition, this database also serves as a platform to visualise and analyse ‘omics’ data. Furthermore, the overall topology of metabolic networks provides insight into the properties of the network, whereas flux analysis permits phenotype predictions at the metabolic level to guide metabolic engineering. The ocsESTdb database will supply a model to derive new non-trivial hypotheses for exploring plant metabolism. Integration of large EST sequences, metabolic pathways and metabolic network data during oil crop seed development gives us insights into the comparative metabolic networks and their difference between green and non-green oilseeds responsible for the synthesis and metabolism of seed oil.

## Availability and requirements

The database is freely available at: http://www.ocri-genomics.org/ocsESTdb/. All data sets are free to use and can be downloaded via the Web interface. There are no restrictions on use of the database or all stores of data sets.

## Additional file

Additional file 1:
**The connection matrix of the enzyme reaction to the newer version of KEGG Ligand (Status August 2009).**
